# Expression of hMLH1 and hMSH2 proteins in ameloblastomas and tooth germs

**DOI:** 10.4317/medoral.22210

**Published:** 2018-02-25

**Authors:** Ronell Bologna-Molina, Vanesa Pereira-Prado, Celeste Sánchez-Romero, Gabriel Tapia-Repetto, Sandra Soria, Marcela Hernandez, Rogelio Gónzalez-Gónzalez, Nelly Molina-Frechero, Toshinari Mikami

**Affiliations:** 1 Molecular Pathology Area, Faculty of Dentistry, Universidad de la Republica, Montevideo, Uruguay; 2 Oral Pathology Section, Department of Oral Diagnosis, Piracicaba Dental School, University of Campinas (UNICAMP), Piracicaba, Brazil; 3 Histology Area, Faculty of Dentistry, Universidad de la Republica, Montevideo, Uruguay; 4 Department of Pathology and Oral Medicine, Faculty of Dentistry, Universidad de Chile, Santiago, Chile/ Dentistry Unit, Faculty of Health Sciences, Universidad Autónoma de Chile, Santiago, Chile; 5 Oral Pathology and Medicine Department, Universidad Juárez del Estado de Durango, México; 6 Health Care Department, Universidad Autónoma Metropolitana, México; 7 Division of Anatomical and Cellular Pathology, Department of Pathology, Iwate Medical University, Morioka, Japan

## Abstract

**Background:**

Mismatch repair proteins (MMRPs) are a group of nuclear enzymes that participate in the repair of base mismatches that occur during DNA replication in all proliferating cells. The most studied MMRPs are hMSH2 and hMLH1, which are known to be highly expressed in normal tissues. A loss of MMRPs leads to the accumulation of DNA replication errors in proliferating cells. Ki-67 is a biomarker regarded to be the gold-standard tool for determining cell proliferation by immunohistochemical methods. The aim of this study was to investigate the immunohistochemical expression of hMLH1, hMSH2 and Ki-67 proteins in ameloblastomas and tooth germs, to contribute to the understanding of the development of this odontogenic neoplasm.

**Material and Methods:**

Immunohistochemical assays to determine the presence of proteins hMSH2, hMLH1 and Ki-67 were performed in 80 ameloblastomas (40 solid and 40 unicystic) and five tooth germs.

**Results:**

Unicystic ameloblastomas showed higher MMRP expression (hMLH1: 62.5 ± 43.4; hMSH2: 83.3 ± 47.8) than did solid ameloblastomas (hMLH1: 59.4 ± 13.5; hMSH2: 75.8 ± 40.2). Additionally, the cell proliferation index assessed by Ki-67 was inversely proportional to the expression of MMRP. Comparison between tooth germs and ameloblastoma revealed significantly higher expression of hMLH1, hMSH2 and Ki-67 in tooth germs (*p*=0.02).

**Conclusions:**

The differences of MMRP and Ki-67 immunoexpression between ameloblastomas and tooth germ suggest that alterations in the MMRP mechanisms could participate in the biological behavior of ameloblastomas.

** Key words:**hMLH1, hMSH2, Ki-67, Ameloblastomas, Tooth germs.

## Introduction

The frequency of DNA mutations is minimized by repair systems that recognize and correct errors that occur during DNA replication; this process is very important for cell survival. Repair systems can recognize mispaired, altered, or missing bases in DNA, as well as other structural alterations of the double helix (1). Repair genes are classified based on the pathway they use to reverse or avoid damage to DNA (recombination repair, base excision repair, nucleotide excision repair and mismatch excision repair).

The mismatch repair (MMR) system in human cells is composed of at least six genes (msh2, mlh1, msh3, pms1, hpms2 and gtbp/msh6); the mismatch repair proteins (MMRPs) are very important for recognition and repair of mispairing and slippage errors in DNA synthesis. MMRPs are a group of nuclear enzymes that participate in the repair of base-base mismatches in all proliferating cells during DNA replication (2). The proteins form complexes (heterodimers) that bind to areas of abnormal DNA and initiate its removal. A mismatch is detected by two complexes, hMSH2-GTBP/hMSH6 and hMSH2-hMSH3 heterodimers, after interaction with hMLH1-hPMS2 heterodimers. Then, an endonuclease is activated, which cuts the newly synthesized DNA strand that contains the mutation (3,4). In this system, the hMSH2 and hMLH1 proteins have been the most studied, and it is known that they are highly expressed in normal tissues, especially in rapidly renewing human cells, such as basal cells of the epidermis and the oral mucosa (5,6). A loss of MMRPs allows the accumulation of replication errors in the DNA of proliferating cells, particularly in genome areas with short repetitive nucleotide sequences, a phenomenon known as microsatellite instability (MSI) (7). hMLH1 is one of the major mismatch DNA repair genes, and its inactivation increases MSI in a variety of human cancers, including head and neck squamous cell carcinoma (HNSCC) (8). Loss of the antimutagenic and antirecombinogenic effects of the mismatch repair system may contribute to tumorigenesis by elevating both the rate of mutation (base substitutions and frame shifts) and mitotic recombination (loss of heterozygosity and chromosomal rearrangements) (9). Mutations in these genes have been linked to hereditary nonpolyposis colon cancer; and they also occur in a variety of sporadic cancers (10).

The correlation between the loss of MMRP expression with the severity of epithelial dysplasia in oral potentially malignant lesions and with the degree of differentiation in oral squamous cell carcinoma has been established in previous studies (11,12). The expression patterns and roles of MMRP in ameloblastoma are not clear at all; there have only been two studies about immunoexpression of MMRP in ameloblastomas. The first focused on hMLH1 and hMSH2 (13), and the second studied hMSH2, hMSH3 and hMSH6 (14). However, the results and conclusions varied in several aspects, which must be clarified with additional studies.

Because proliferation mechanisms are dysregulated in neoplasms, Ki-67 can indicate tumor aggressiveness by allowing assessment of the proliferation index, which has been associated with expression of the MMR complex (15,16).

Since the development of monoclonal antibodies against the hMSH2 protein by Leach *et al.* in 1996, several studies have been performed to determine the utility of immunohistochemical detection of MMRP (17). The aim of this study was to investigate the expression of hMLH1, hMSH2 and Ki-67 proteins in ameloblastomas and tooth germs with immunohistochemical methods to contribute to the understanding of the development of this odontogenic neoplasia.

## Material and Methods

We examined the protein expression patterns of hMSH2, hMLH1 and Ki-67 by immunohistochemistry in 80 ameloblastomas (40 solid and 40 unicystic) and in five tooth germs from the Molecular Pathology Area and Histology laboratory from Facultad de Odontologia, Universidad de la Republica, Uruguay. The research project was approved by Ethics Committee number 091900/000120/06. The authors have read the Declaration of Helsinki and have followed its guidelines in this investigation. All tumors were diagnosed by histopathological evaluation of surgical specimens and were classified according to the 2017 WHO head and neck tumor classification standards (18). All specimens were fixed in formalin, pH 7.2, and embedded in paraffin wax following standard procedures. Two-μm sections were mounted on silanized slides and deparaffinized in an oven on 45°C for 30 minutes. Then, the sections were deparaffinized with xylene and subsequently rehydrated with a graded ethanol series and water. Sections were immersed in 10 mM sodium citrate buffer (pH 6.2) and placed in a pressure cooker for microwave antigen retrieval for 5 minutes. Endogenous peroxidase activity was blocked by incubation with 0.9% H2O2 for 5 minutes and then buffered with PBS (pH 7.4). Mouse monoclonal primary antibodies against hMLH1 (Clone ES05), hMSH2 (clone Clone FE11) and Ki-67 (clone MIB-1) antibodies were diluted 1:100, and incubated on sections for 45 minutes. After incubation, all slides were exposed to avidin-biotin complex and horseradish peroxidase reagents (LSAB Kit; Dako Cytomation, Carpinteria, CA) and diaminobenzidine tetrahydrochloride (DAB; Sigma-Aldrich, St Louis, MO). Finally, the sections were counterstained with hematoxylin, dehydrated in graded alcohol and cleared with xylene. As a positive control, sections of human tonsil were used, whereas negative controls were obtained by omitting the specific primary antibody. In tooth germs the presence of the proteins studied was evaluated in the enamel organ.

The index of nuclear positivity was obtained using our previously described method (19) and was expressed as the percentage of positive cells per square millimeter of tissue.

To detect mutations in hMLH1 and hMSH2, Next Generation Sequencing was performed. In this research, we used HAM (Human Ameloblastoma) cell lines (HAM1, HAM2 and HAM3), which were established from the same ameloblastoma case of the mandible (20).

The results were analyzed by χ2 tests and Spearman correlation using SPSS 16.0 statistical software (SPSS Professional Statistics, SPSS Inc., Chicago, IL) and were considered significant when *p*≤0.05.

## Results

**Figure 1:**
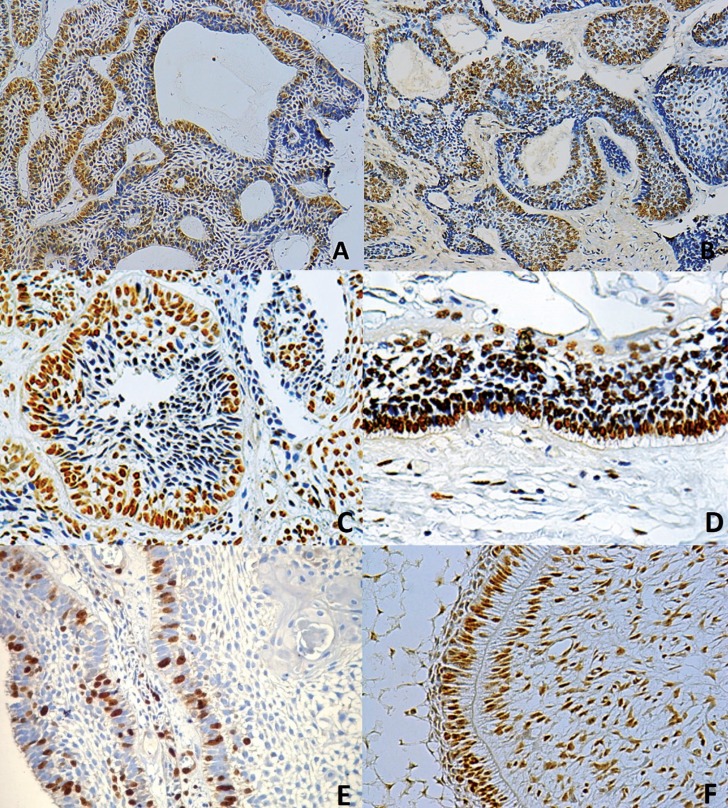
Immunohistochemical localization of hMLH1 (A,C), hMSH2 (B,D) and Ki-67 (E) proteins in different ameloblastomas. Immunohistochemical expression of hMSH2 protein in tooth germ (F) (immunoperoxidase, 20x).

The localization of the hMSH2 and hMLH1 staining was exclusively nuclear. In ameloblastomas, immunoexpression was observed in epithelial tumor nests at the outer basal and columnar cells, similar to ameloblasts, and in the inner angular cells resembling the stellate reticulum (Fig. 1 A-D). In enamel organ of the tooth germ, immunoexpression was observed in enamel epithelium as well as in the stellate reticulum. No correlation between clinical data (age, sex, localization and tumor size) and MMRPs was found.

When MMRPs were evaluated in unicystic ameloblastomas, they showed higher expression (hMLH1: 62.5 ± 43.4; hMSH2: 83.3 ± 47.8) than did solid ameloblastomas (hMLH1: 59.4 ± 13.5; hMSH2: 75.8 ± 40.2). However, expression of Ki-67 was inversely proportional to the expression of MMRP, which indicates that lower MMRP correlated with a higher Ki-67 index (Fig. 1 E).

Comparison of tooth germs to ameloblastomas revealed significantly higher expression of MMRP and Ki-67 in tooth germs (*p*=0.02); (Fig. 1 F).

Expression levels of Ki-67, hMLH1 and hMSH2 in ameloblastomas and tooth germs are reported in  Table 1. Spearman correlation values are shown in  Table 2. No mutations in hMLH1 and hMSH2 were found by Next Generation Sequencing in ameloblastoma cell lines, (data not shown).

**Table 1:**
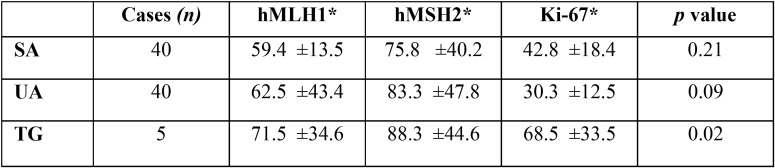
Levels of hMLH1, hMSH2 and Ki-67 expression in ameloblastomas and tooth germs. TG: tooth germ, UA: unicystic ameloblastoma, SA: solid ameloblastoma. *: percentage and standard deviation.

**Table 2:**
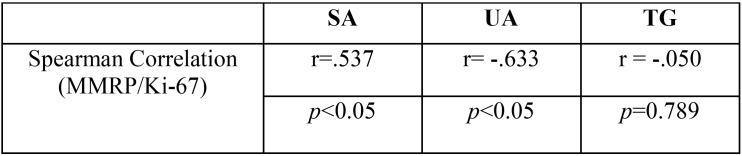
Spearman correlation between mismatch repair proteins (MMRP) and Ki-67 in solid ameloblastoma (SA), unicystic ameloblastoma (UA) and tooth germ (TG).

## Discussion

Our results indicate that ameloblastomas expressed lower levels of both hMSH2 and hMLH1 proteins compared to normal tissue (tooth germs), we also observed differences between the expression of these proteins by immunohistochemical analysis in solid and unicystic variants.

Castrilli *et al.* reported for the first time the presence of MMRP in ameloblastomas, and recently Amaral-Silva *et al.*, who also included tooth germ samples in their study (13,14). Consistent with these results, we found exclusive nuclear localization of hMLH1 and hMSH2. In the first study, hMLH1 and hMSH2 immunostaining was observed predominantly in the outer layer of tumor nests, whereas in the present study, variable staining was observed in both the outer/ameloblastic layer and stellate inner cells of tumoral nests, similarly to the second study, which also showed that MMRP are decreased in ameloblastomas in comparison with tooth germ, as we found in this study.

Regarding the association of MMRP with pathogenesis or clinical features of ameloblastoma, the first study suggested that hMLH1 and hMSH2 do not have a specific function related to the development and behavior of this tumor (13). In contrast, the latest study found a correlation between the simultaneous overexpression of three MMRP and tumor recurrence (14).

The MMR system is responsible for removing mismatched bases caused by spontaneous damage, amination of bases, oxidation, methylation and errors in the replication or recombination processes (1).

The importance of the MMR system is to maintain genomic stability and reduce mutations during replication. Individuals with related MMR mutations often present syndromes with tumor predisposition and several types of cancer (21).

Defects in this MMR pathway result in an increased rate of mutation or MSI, which leads to defects in genes that regulate cell proliferation and death (22), thereby increasing susceptibility to cancer. Therefore, proper activity of this system is essential to preserve normal cellular functions.

The immunoexpression of hMLH1 and hMSH2 has been found in various normal tissues (23), and their roles have been correlated with normal reparative functions. It is expected that normal tissues express these two proteins, as with any normal DNA repair system. In fact, in the present study, we found that normal dental tissue (tooth germs) shows higher expression of hMLH1 and hMLH2 compared with neoplastic tissue (ameloblastoma). Because the function of both proteins is to repair DNA damage, it is not surprising that they are present in cells of constantly renewed tissues, such as oral keratinocytes (5), and in this case, in embryonic cells forming dental tissues.

On the other hand, we found differences between the expression of MMRP and the histological type of ameloblastoma. The unicystic type presented higher expression of hMLH1 and hMSH2 than solid ameloblastoma, suggesting that the decrease of MMRP in solid ameloblastoma may play a role in its more aggressive course by altering the repair mechanisms. Gene mutations affect protein expression in different ways. Most mutations in hMSH2 are protein truncating; consequently, no hMSH2 is detected by immunohistochemistry in these cases (24). Decreased expression of hMLH1 and hMSH2 in ameloblastomas could be result of a gene mutation; our group analyzed gene mutation of hMLH1 and hMSH2 in a HAM cell lines by next-generation sequencing, however, no mutations were detected (data not shown). This result suggested that gene mutations are not the direct cause of the decreased expression of hMLH1 and hMSH2 in ameloblastoma.

Modification in the gene expression without alteration in the DNA sequence can be due to epigenetic changes, which are potentially inherited but also reversible. The main epigenetic mechanisms include DNA methylation and histone acetylation or deacetylation. Recently, evidence for frequent MSH2 hypermethylation in Lynch Syndrome tumors with MSH2 deficiency was reported (25,26). In hereditary cancers, somatic mutation is the main mechanism for hMLH1 gene inactivation; in sporadic cancers, epigenetic hMLH1 silencing by promoter hypermethylation appears to be involved (27,28). For HNSCC, it was previously reported that hypermethylation of the hMLH1 promoter is a mechanism of gene inactivation, and therefore, loss of protein expression (27).

Another possible pathway to explain our findings is that the decreased levels of hMLH1 and hMSH2 protein expression is affected by the regulation of mRNA by miRNAs. It is well known that very small RNAs (~22 base) or miRNAs regulate gene expression by base pairing with complementary untranslated sequences in target mRNA, thereby regulating gene expression. miRNAs present variations between malignant and normal cells (29,30). Further studies need to be performed in this field to confirm or discard this theory for ameloblastomas.

Interestingly, we observed that as hMLH1 and hMSH2 expression was higher, the Ki-67 index decreased in ameloblastomas; however, this relationship was not present in tooth germs, which had high levels of hMLH1, hMSH2 and Ki-67. This suggests different implications for MMRP in tumorigenic mechanisms and in the development of normal tissues.

In contrast to the study by Amaral-Silva *et al.*, in which no correlation between MMRP and Ki-67 was found in ameloblastomas, we observed a negative correlation between MMRP and Ki-67, which probably means that a lack of proteins associated with DNA repair can be associated with an increase in cell proliferation and therefore tumoral behavior (31). This increase of proliferation and decrease of DNA repair proteins, observed markedly in solid ameloblastomas, may contribute to the higher aggressiveness of this variant. On the other hand, the higher expression of MMRP and Ki-67 in normal odontogenic tissue (tooth germs) is important, considering that it is an embryonic tissue with high proliferation, which requires more DNA repair system activity.

In summary, we detected a non-mutational decrease of hMLH1 and hMSH2 protein levels, along with an increased proliferation rate in ameloblastomas that may be associated with tumor behavior (aggressiveness) and lead to DNA susceptibility to replication errors. Normal odontogenic tissue (tooth germ) presented a high proliferation index and higher expression of hMLH1 and hMSH2, which are physiologically necessary to repair DNA in all normal proliferative tissues, including embryonic cells. The data obtained in this work provide complementary novel information that contributes to the understanding of the roles of MMRPs (hMSH2 and hMLH1) in the biological behavior of ameloblastomas through interactions with Ki-67 expression.

## Conclusions

The results suggest that changes in the correlations between immunoexpression of MMRPs and Ki-67 could be related to the biological behavior of ameloblastomas and represent a physiologic mechanism in tooth germs.
